# CRISPR/Cas-Mediated Targeted Mutagenesis in *Daphnia magna*

**DOI:** 10.1371/journal.pone.0098363

**Published:** 2014-05-30

**Authors:** Takashi Nakanishi, Yasuhiko Kato, Tomoaki Matsuura, Hajime Watanabe

**Affiliations:** Graduate School of Engineering, Osaka University, Suita, Osaka, Japan; Oxford Brookes University, United Kingdom

## Abstract

The water flea *Daphnia magna* has been used as an animal model in ecology, evolution, and environmental sciences. Thanks to the recent progress in *Daphnia* genomics, genetic information such as the draft genome sequence and expressed sequence tags (ESTs) is now available. To investigate the relationship between phenotypes and the available genetic information about *Daphnia*, some gene manipulation methods have been developed. However, a technique to induce targeted mutagenesis into *Daphnia* genome remains elusive. To overcome this problem, we focused on an emerging genome editing technique mediated by the clustered regularly interspaced short palindromic repeats/CRISPR-associated (CRISPR/Cas) system to introduce genomic mutations. In this study, we targeted a functionally conserved regulator of eye development, the *eyeless* gene in *D. magna*. When we injected Cas9 mRNAs and *eyeless*-targeting guide RNAs into eggs, 18–47% of the survived juveniles exhibited abnormal eye morphology. After maturation, up to 8.2% of the adults produced progenies with deformed eyes, which carried mutations in the *eyeless* loci. These results showed that CRISPR/Cas system could introduce heritable mutations into the endogenous *eyeless* gene in *D. magna*. This is the first report of a targeted gene knockout technique in *Daphnia* and will be useful in uncovering *Daphnia* gene functions.

## Introduction

The water flea *Daphnia magna* is a planktonic crustacean ubiquitously found in the fresh water environment. It has been used as a model organism in ecology and toxicology because it is sensitive to artificial chemicals and environmental changes [1]. Moreover, researchers find it interesting that *Daphnia* can switch their reproduction mode from asexual to sexual in response to environmental stimuli [2]. Recent progress in genomics involved analyses of expressed sequence tags (ESTs) [3] and the draft genome sequence of *D. magna*. In addition, the genome sequence of a related organism, *D. pulex*, has recently been completed [4]. Therefore, a vast amount of genetic information on *Daphnia* is now available. To investigate the relationship between available genetic information and phenotypes, gene manipulation tools such as RNA interference (RNAi) and non-homologous integration with plasmid DNA have been developed in *D. magna* [5], [6]. However, there is still no technique to induce inheritable targeted gene disruptions.

**Figure 1:**
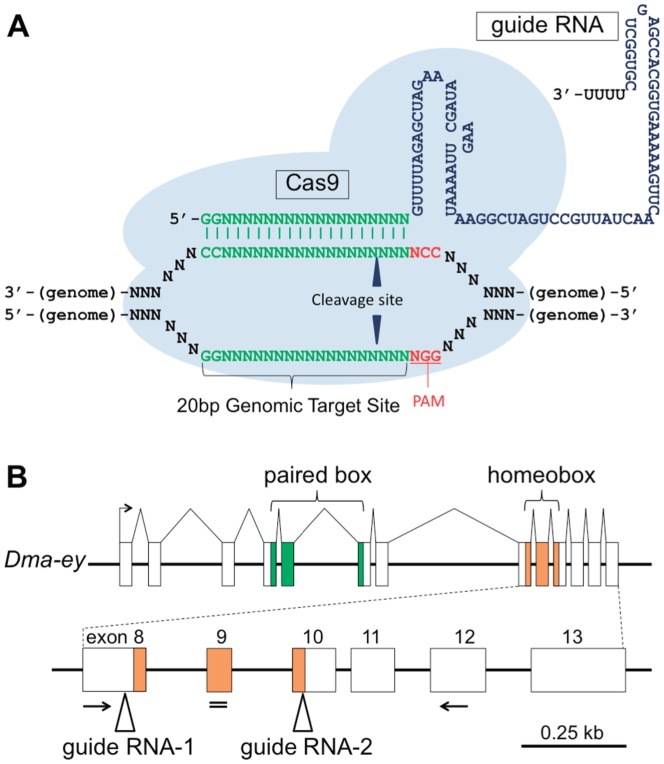
A target gene for the CRISPR/Cas-based targeted mutagenesis: *D. magna eyeless* (*Dma-ey*) gene. (**A**) Schematic illustration of the CRISPR/Cas system. The guide RNA (gRNA) binds to the 20-bp genomic target site with its complementary sequences (green). The genomic target site requires the PAM sequence (NGG; red) immediately downstream of its 3′ side. The latter sequences of gRNA (blue) interact with Cas9 nuclease (light blue spheres). Cleavage sites are shown by triangles. (**B**) Schematic illustration of *Dma-ey* locus. *Dma-ey* gene putatively consists of 13 exons (shown by open boxes). DNA-binding domain-encoding regions are colored in green (paired box) and orange (homeobox). Primers designed for RT-PCR are indicated by arrows. siRNA was targeted in a site shown by a double line. gRNA-targeted sites are depicted by triangles.

The clustered regularly interspaced palindromic repeats/CRISPR-associated (CRISPR/Cas) system is a recently developed tool to induce targeted mutagenesis. CRISPR/Cas was initially identified as the bacterial immune system to bacteriophages [7]. In this system, a CRISPR RNA (crRNA) interacts with a transactivating CRISPR RNA (tracrRNA) and forms the tracrRNA:crRNA duplex, which acts as a guide RNA (gRNA) that directs the endonuclease Cas9 to its cognate target DNA and induces double-strand breaks (DSBs) [8]. Importantly, the cleavage site is often imperfectly repaired by the error-prone non-homologous end-joining (NHEJ) mechanism, resulting in gene disruptions through the introduction of small insertions or deletions (in-dels). Recent studies reported that a single chimeric RNA, a crRNA fused with a tracrRNA, could also function as a gRNA [8]. The approximately 20 bp targetable sites are limited by the requirement for the protospacer adjacent motif (PAM; 5′-NGG-3′) at their 3′ end [8]. Further constraint that target sites start with a GG dinucleotide is often required when gRNAs are synthesized i*n vitro* by T7 polymerase. However, recent studies suggested some alternatives to circumvent the latter limitation without significant reduction of cleavage efficiency [9]–[11].Therefore, to induce targeted mutagenesis, co-expression of the customized gRNA with the Cas9 nuclease has been used in various organisms [9]–[25] (Figure 1A). Compared with the other targeted mutagenesis tools such as zinc finger nucleases (ZFNs) and transcription activator-like effector nucleases (TALENs), the experimental design of CRISPR/Cas is remarkably simple and rapid [26]. However, nobody has applied these emerging techniques to *Daphnia*.

The mammalian *pax6* gene ortholog is a functionally conserved regulator for eye development that has been conserved from invertebrates to vertebrates. It encodes a transcription factor with two DNA-binding domains: paired box and homeobox domains. In *Drosophila*, a mammalian *pax6* homolog was originally mapped in the *eyeless* (*ey*) locus [27], whose mutants show abnormal eye morphogenesis, resulting in complete or partial loss of eye as well as mutations in the mouse *pax6* gene [27]–[31]. Animals with abnormal eyes could be easily distinguished from normal ones in appearance, suggesting that *pax6* ortholog would be a useful model target gene for targeted mutagenesis as previously used for TALEN-mediated mutagenesis [32].

In the present study, *D. magna ey* gene was partially cloned and its role in eye development was confirmed by RNAi, thus prompting us to use *ey* as a target for CRISPR/Cas-mediated mutagenesis. We showed that the CRISPR/Cas system efficiently introduces heritable in-del mutations into the *ey* loci of the *D. magna* genome, resulting in deformations of the compound eye. In addition, undesired mutations (off-target mutations) were not detected in the genomes of the *ey*-deficient daphniids. Thus, we concluded that the CRISPR/Cas system is a powerful tool for modifying targeted genomic sites and can facilitate studies on the functional genomics of *D. magna*.

## Results

### Functional analyses of *D. magna eyeless* (*Dma-ey*) gene

To utilize the *ey* gene as a marker gene for targeted mutagenesis, we searched for mammalian *pax6* orthologs in the *D. magna* genome and found the *ey* gene in addition to its paralog, *twin of eyeless* (*toy*) gene, both of which are conserved among several arthropods [33]. We named *D. magna ey* as *Dma-ey* in this study. By using the *D. magna* genome database, the *Dma-ey* gene was predicted to consist of 13 exons (Figure 1B). Reverse transcription-PCR (RT-PCR) of the *Dma-ey* gene using a primer set encompassing the homeobox as well as sequencing of the PCR fragments revealed that *Dma-ey* is expressed in *D. magna* (Figure S1).

**Figure 2:**
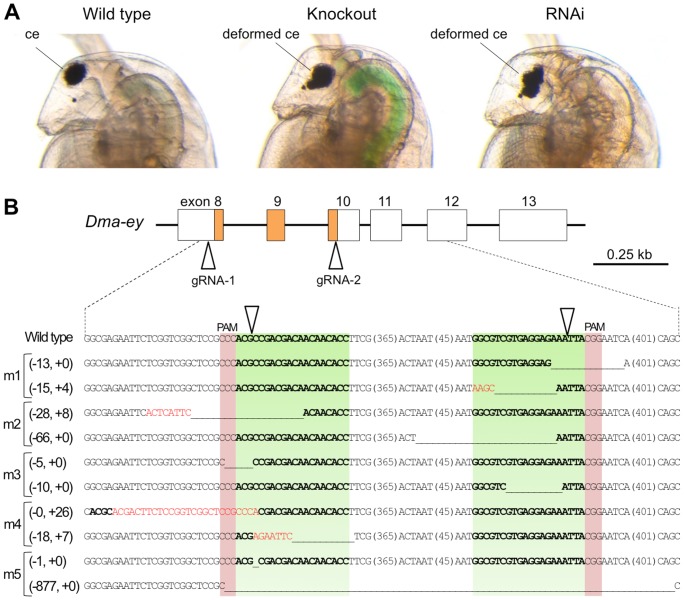
Knockout of *Dma-eye* gene. (**A**) Typical phenotypes of *Dma-ey* deficient daphniids. The images to the left, center and right show the lateral head parts of the wild-type daphniid, *Dma-ey* knocked-out daphniid by the CRISPR/Cas system, and *Dma-ey* RNAi daphniid, respectively. The knocked-out individual is “mutant #5 (m5)” described in Figure 2B. Ventral side is left. ce: compound eye. (**B**) Genome sequences of established *Dma-ey*-knocked out mutant lines around gRNA-targeted sites. A part of the exon-intron structure of the *Dma-ey* gene including the homeobox colored in orange is shown above. In the alignment, the top line in the below alignment represents the wild-type *Dma-ey* sequence, and subsequent lines show sequences of five mutant alleles (see Table 1). The target sites for gRNAs are indicated in green, PAM in red, and cleavage site by triangles. The length (base pairs) of each in-del mutation is written in the left of each sequence (-, deletions; +, insertion). In each mutant sequence, deletion is indicated by underbars, insertions by red letters, sequences corresponding to the wild-type targeted sequences by bold letters, and the length (base pairs) of an abbreviated sequence within a parenthesis.

Next, we tested whether the *Dma-ey* gene is required for compound eye development by RNAi. We designed siRNA within the homeobox of *Dma-ey*. A compound eye in a wild-type daphniid is located at the anterior parietal edge and roughly spherical in shape (Figure 2A, left), whereas daphniids injected with 100 µM siRNA had deformed compound eyes, which were located at the inner side and could not build a precise spherical shape (Figure 2A, right). Afterward, we call this phenotype as deformed eye in this study. Taken together, our results indicated that the *eyeless* gene is functionally conserved in *D. magna*, suggesting that it would be a useful marker gene for subsequent knockout experiments using the CRISPR/Cas system.

### *Dma-ey* gene disruption by CRISPR/Cas system

To test whether the CRISPR/Cas system could induce targeted mutagenesis in *D. magna*, we attempted to introduce in-del mutations into the homeobox domain of the *Dma-ey* locus. Previous reports described *ey*-deficient *Drosophila* flies whose mutated *ey* allele lacked all C-terminal domains including the homeobox domain [31]. Thus, we hypothesized that *Dma-ey*-deficient daphniids can be generated by inducing frameshifts at an earlier part of the homeobox domain. To target the homeobox region, we used two gRNAs, gRNA-1 and gRNA-2, which were designed to bind the sense strand from exon 8 and the anti-sense strand from exon 10, respectively (Figures 1B and 2B). Because a previous report suggested that co-injection of multiple gRNAs increased mutation efficiency [17], we coinjected two gRNAs together with the Cas9 mRNA that contained the untranslated regions (UTRs) of *D. magna vasa* (*Dmavas*) gene, which is exclusively expressed in *D. magna* germ cells [34]. We tested three and two different concentrations of Cas9 mRNA (500; 1,000; 2,000 ng/µL) and gRNAs (50; 150 ng/µL each), respectively (Table 1). At the first instar juvenile stage, 61–78% of the injected embryos survived. In addition, as expected, 18–47% of the surviving juveniles developed deformed eye phenotypes without significant dose dependency, suggesting that the CRISPR/Cas system worked in *D. magna* (Table 1).

**Table 1:** Mutation frequencies induced by microinjection using various concentrations of Cas9 mRNA/gRNA mix.

RNA concentration (ng/µL)		Embryos	Juveniles		Adults	
gRNA mix	Cas9 mRNA	Injected	Surviving	Deformed eye	Surviving	Founder lines
50 each	500	77	59/77 (77%)	28/59 (47%)	49/77 (64%)	*4/49 (8.2%)
	1,000	121	90/121 (74%)	16/90 (18%)	81/121 (67%)	5/81 (6.2%)
	2,000	113	75/113 (66%)	29/75 (39%)	61/113 (54%)	0/61 (0%)
150 each	500	98	70/98 (71%)	19/70 (27%)	60/98 (61%)	2/60 (3.3%)
	1,000	86	67/86 (78%)	20/67 (30%)	60/86 (70%)	*2/60 (3.3%)
	2,000	64	39/64 (61%)	8/39 (21%)	38/64 (59%)	*1/38 (2.6%)

*Mutant lines subjected to sequencing of *Dma-ey* loci. In Figure 2B, two mutants named m1 and m2 were from 4 mutants injected with 50 ng/µL each of gRNA and 500 ng/µL Cas9 mRNA (50 each, 500), m3 and m4 from 2 mutants (150 each, 1,000), and m5 from 1 mutant (150 each, 2,000).

After the injected animals matured, we counted the number of adults producing deformed eye G1 progenies ( =  founder G0 animals) and normal eye G1 progenies, respectively, to calculate the efficiency in inducing heritable mutations. The founder G0 animals were generated at a rate of 2.6–8.2% of the surviving adults, excluding the case when 50 ng/µL each of gRNA-1 and gRNA-2 were injected with 2,000 ng/µL Cas9 mRNA (Table 1 and Figure 2A). The highest efficiency was achieved by injecting 50 ng/µL each of gRNA-1 and gRNA-2 with 500 ng/µL Cas9 mRNA. Of the 14 founder animals, 13 founders produced deformed eye progenies which died within a week. The lethality of *Dma-ey* knockout mutants was consistent with the previous report on *Drosophila* [31]. However, the remaining founder animal produced viable deformed eye progenies and the phenotype was observed over generations. The difference of viability between mutant lines is discussed in detail (see Discussion). In sum, these results suggest that the CRISPR/Cas system worked not only in somatic cells but also in germ line cells.

To investigate how in-del mutations were introduced into the *Dma-ey* loci, we collected deformed eye G1 progenies from 5 different founder lines and extracted their genomic DNAs respectively (Table 1). Genomic PCR products encompassing the gRNA-targeted sites were cloned and sequenced. All of the deformed eye G1 progenies had biallelic in-del mutations around gRNA-targeted sites (Figure 2B). In contrast, monoallelic in-del mutations were found in normal eye G1 progenies (data not shown). Thus, we concluded that the CRISPR/Cas system could induce heritable mutations in the endogenous *Dma-ey* locus of *D. magna*.

### Evaluation of off-target effects in deformed eye mutants

We further analyzed if *Dma-ey*-deficient mutants had undesired mutations (off-target mutations). Pioneering works suggested that genomic sites have mismatches fewer than 5 bp with PAM (NGG at 3′ end) sequence could be cleaved ( =  potential off-target sites) [19], [35]. By using a BLASTn search on the *D. magna* genome database, we looked for potential off-target sites and found that gRNA-1 had 4 potential off-target sites, whereas gRNA-2 did not (Table 2). To test if *Dma-ey*-deficient mutants have off-target in-del mutations, we designed a primer set to amplify each potential off-target site of gRNA-1 and performed PCRs using genomic DNAs from 5 different G1 mutants subjected to previous analyses of in-del mutations in the *Dma-ey* locus. Consequently, no off-target mutation was observed. These results suggested that the CRISPR/Cas-mediated targeted mutagenesis approach was highly specific.

**Table 2:** Potential off target sites of gRNA-1.

	On/off target sites (5′-3′)	Locations	Annotations	Mismatches
On target	GGTGTTGTTGTCGTCGGCGTggg	exon	eyeless	-
Off target	GGTGTTG**G**TGTCGTCGGCGTcgg	exon	protocadherin fat 2 precursor	1 bp
	G**T**TG**G**TGTTGTCGTCGGC**A**Tcgg	exon	cytochrome c oxidase	3 bp
	**AT**TG**G**TGTTGTCGTCGGC**A**Tcgg	exon	cytochrome c oxidase	4 bp
	**T**G**C**G**CC**GTTG**A**CGTCGGCGTtgg	intronic	-	5 bp

Genomic sequences of on/off target sites (uppercase) with PAM (NGG, lowercase), their locations, annotations, and the number of base pair differences are noted. Bold letters exhibit mismatched nucleotides. bp: base pairs.

## Discussion

Here, we described a simple, rapid, and efficient technique for inducing target mutagenesis into an endogenous locus of the *D. magna* genome by using a CRISPR/Cas system, which will enable us to perform high-throughput functional analyses of *D. magna* genes. This system would contribute to overcoming two limitations in previous *Daphnia* reverse genetics studies using RNAi by microinjection of dsRNAs into eggs [5]: (1) incapability to induce null phenotypes and (2) transient nature of gene manipulation. The knockout *Daphnia* lines allow us to observe the loss-of-function phenotypes throughout life cycle, which will undoubtedly advance our understanding of *D. magna* gene functions.

We coinjected pairs of gRNAs, gRNA-1, and gRNA-2, which targeted exons 8 and 10 of *Dma-ey*, together with Cas9 mRNA. We found that all five *Dma-ey* G1 mutants with deformed eyes have biallelic mutations. This simple and efficient induction of biallelic mutations seems to be beneficial for researchers studying *Daphnia* that usually produce parthenogenetic females. For crossing to establish homozygous mutants in *Daphnia*, we have to induce the production of males and sexual females that lay haploid eggs by stimulating the parthenogenetic females with environmental cues such as shortened photoperiod, lack of food, and/or increased population density, which makes the crossing procedure laborious and time consuming. Ability of the CRISPR/Cas system to induce biallelic mutations would significantly improve the genetics of parthenogenetic organisms, including *Daphnia*.

In the current study, no founder animals were observed when we used 2,000 ng/µL of Cas9 mRNA and 50 ng/µL of each gRNA for injection. One possible explanation for this result is the aggregation of free abundant Cas9 proteins which prevents them from forming a complex with gRNAs. This interpretation seemingly corresponds with our data since we could establish one founder animal by injection of the same concentration of Cas9 mRNA with a higher concentration of gRNAs (see Table 1). Although the reason is not clear based on our current data, the proportion of Cas9/gRNA used should be important for the activity of CRISPR/Cas systems.

Of the 14 deformed eye mutants established in this study, one was viable. Biallelic *Dma-ey* mutations should give rise to lethality because previous reports described that homozygous mutation in mammalian *pax6* and its homolog *ey* were lethal to mice and flies, respectively [29], [31]. Our sequence data showed that the viable mutant lost the 877 bp region including the entire homeobox domain on the *Dma-ey* allele but it retained the correct reading frame of the remaining C-terminal region (Figure 2B, m5), whereas coding frames of the other sequenced alleles were shifted, hence all the sequences from the homeobox domain to the C-terminal region were lost (Figure 2B. m1 to m4). This might account for the difference in the mutants' viability. Previous works described the PAX6 protein as having a conserved C-terminal domain which, follows homeobox domain, is rich in proline (P), serine (S), and threonine (T) residues, and which mediates activation of PAX6 protein via phosphorylation [36], [37], [38]. Therefore, the presence of the C-terminal activation domain on the 877 bp deleted *Dma-ey* allele might contribute to the viability of the deformed eye mutant *D. magna*.

Of the 10 alleles from five deformed eye mutants analyzed in this study, nine had indel mutations in one of the two target sites (Figure 2B). The patterns of these mutations were consistent with those induced by Cas9-based cleavage in the other animals. Interestingly, one allele had an 877-bp deletion spanning both target sites, suggesting the possibility that concurrent DSBs at two distantly target sites for gRNAs induced this large deletion, as reported in previous studies [9], [12], [17], [22].

CRISPR-based genome engineering will also provide novel approaches to integrate foreign DNAs into the *D. magna* genome. DSB sites are predominantly repaired by either NHEJ or homologous recombination (HR) [39]. Double-strand cleavage has been shown to facilitate the rate of homologous gene targeting at the cleaved site [40]. Successes of targeted knock-ins have been reported through the co-introduction of CRISPR components and exogenous DNAs such as plasmids or single-strand oligoDNAs (ssODNs) that have a homologous region to the cleavage site [16], [17], [19], [25]. This approach will enable us to induce targeted knock-in of DNA fragments such as integrase-targeting sequences or epitope tag-coding sequences, even in *D. magna*.

Thus, CRISPR/Cas system-mediated genome editing technique described here will definitely accelerate the development of *Daphnia* functional genomics.

## Materials and Methods

### *Daphnia* strain and culture conditions

The *D. magna* strain (NIES clone) was obtained from the National Institute for Environmental Studies (NIES, Tsukuba, Japan) and cultured under laboratory conditions for many generations. To minimize variations in maternal effects that may influence microinjection, we maintained the strain under the following conditions: 120 neonates (under 24 h) were transferred to 5 L of ADaM medium [41] and cultured at 22–24°C, under a light/dark photoperiod of 16 h/8 h. The culture medium was not changed for 3 weeks. Daphniids were fed once a day with 7.5 mg of *Chlorella vulgaris* (Nikkai Center, Tokyo, Japan) and 7.5 mg of baker's yeast (marusanPantry, Ehime, Japan) during the first week; after they matured, their offspring was removed once per day and they were fed 15 mg of *Chlorella* and 15 mg of yeast daily. The addition of yeast helped in maintaining the number of juveniles per clutch constantly higher than that observed when fed with *Chlorella* only (data not shown).

### Construction of RNA expression vectors

To generate the Cas9 expression vector pCS-Dmavas-Cas9, the Cas9 ORF was amplified using the plasmid MLM3613 (Addgene plasmid 42251, [18]) as a PCR template. DNA fragments of the 5′ and 3′ UTRs from the *Dmavas* gene (Accession: AB193324.1) were obtained by PCR using cDNAs of the NIES strain. These PCR products were simultaneously cloned into the downstream region of the SP6 promoter of pCS+ vector using the In-Fusion PCR cloning kit (Clontech, California, USA) [42]. To generate the gRNA expression vector pDR274-Dma-ey, the plasmid DR274 (Addgene plasmid 42250, [18]) was digested with *Bsa*I (NEW ENGLAND BioLabs, Connecticut, USA), followed by dephosphorylation with Antarctic Phosphatase (NEW ENGLAND BioLabs, Connecticut, USA). A pair of *Dma-ey* targeting oligonucleotides was annealed and then ligated into the linearized pDR274 vector using a ligation mix (TaKaRa Bio, Shiga, Japan). The genomic target sites and sequences of the oligonucleotides constructed in this study are listed in Tables S1 and S2. All PCRs in this section were performed with PrimeSTAR (Takara Bio, Shiga, Japan).

### *In vitro* RNA synthesis

siRNAs for knocking down the *Dma-ey* gene were designed by using Block-iT RNAi Designer (Life Technologies, California, USA) and two nucleotides dTdT were added to each 3′ end of the siRNA strand. The sequences of the siRNA are listed in Table S1.

For the syntheses of Cas9 mRNAs, templates with T7 promoter were amplified by PCR from the pCS-Dmavas-Cas9. The primer sequences for the PCR are shown in Table S2. Amplified PCR fragments were subjected to *in vitro* transcription with the mMessage mMachine T7 kit (Life Technologies, California, USA). Poly (A) tails were attached to capped Cas9 RNAs by using a Poly(A) Tailing Kit (Life Technologies, California, USA), following the manufacturer's instructions. The synthesized mRNAs were column purified using mini Quick Spin RNA columns (Roche diagnostics GmbH, Mannheim, Germany), followed by phenol/chloroform extraction, ethanol precipitation, and dissolution in DNase/RNase-free water (Life Technologies, California, USA).

For the syntheses of gRNAs, pDR274-Dma-ey vectors were digested by *Dra*I and purified by phenol/chloroform extraction. *Dra*I-digested DNA fragments were used as templates for *in vitro* transcription with the mMessage mMachine T7 kit, followed by column purification with mini Quick Spin RNA columns, phenol/chloroform extraction, ethanol precipitation, and dissolution in DNase/RNase-free water.

### Microinjection

*In vitro* synthesized RNAs were injected into *Daphnia* eggs according to established procedures [5]. Briefly, eggs were collected from daphniids within 2–3 weeks of age just after ovulation and placed in ice-chilled M4 medium containing 80 mM sucrose (M4-sucrose). The synthesized RNAs were injected through a glass needle with N_2_ gas pressure. The injection volume was approximately 0.2 nL. Finally, an injected egg was transferred into each well of a 96-well plate filled with 100 µL of M4-sucrose. Microinjections were carried out within an hour after ovulation.

### PCR amplification of target loci

To characterize Cas9-induced mutations at the molecular level, target loci were amplified by PCR using genomic DNA extracted from deformed eye daphniids. Genomic DNA was extracted from single daphniids by homogenization in 90 µL of 50 mM NaOH with zirconia beads. The lysate was heated at 95°C for 10 min and then neutralized with 10 µL of 1 M Tris-HCl (pH 7.5). This crude DNA extract was centrifuged at 12,000 rpm for 5 min prior to being used as a template for genomic PCR. The targeted genomic region was amplified by PCR with KOD plus (TOYOBO, Osaka, Japan). The PCR products were analyzed by agarose gel electrophoresis and DNA sequencing. The primers used for PCR and DNA sequencing are listed in Tables S3 and S4.

## Supporting Information

Figure S1 **Results of RT-PCR for** ***Dma-ey*** **and cloned partial cDNA sequence.** (**A**) Electrophoresis of PCR products. M: 100-bp ladder marker (TOYOBO, Osaka, Japan), G: genomic PCR product as positive control of PCR, RT+: RT-PCR product amplified from reverse-transcribed cDNAs, RT-: RT-PCR product amplified from total RNAs without reverse transcription. (**B**) Sequence of partially cloned *Dma-ey* cDNA.
(TIF)

Table S1 **Oligonucleotides for gRNAs and siRNAs.** Lowercase “tt” in sense and antisense oligonucleotides for siRNA means dTdT (see Materials and Methods).
(DOCX)

Table S2 **Oligonucleotides used as** ***in vitro*** **transcription templates.**
(DOCX)

Table S3 **Oligonucleotides used for the verification of cutting sites by sequencing.**
(DOCX)

Table S4 **Oligonucleotides used for verification of off-target mutations by sequencing.**
(DOCX)
