# Supplementary figures and images for: CRISPR/Cas-Mediated Targeted Mutagenesis in Daphnia magna
Source: PLoS One. 2014 May 30;9(5):e98363. doi: 10.1371/journal.pone.0098363 (PMC4039500; doi:10.1371/journal.pone.0098363)

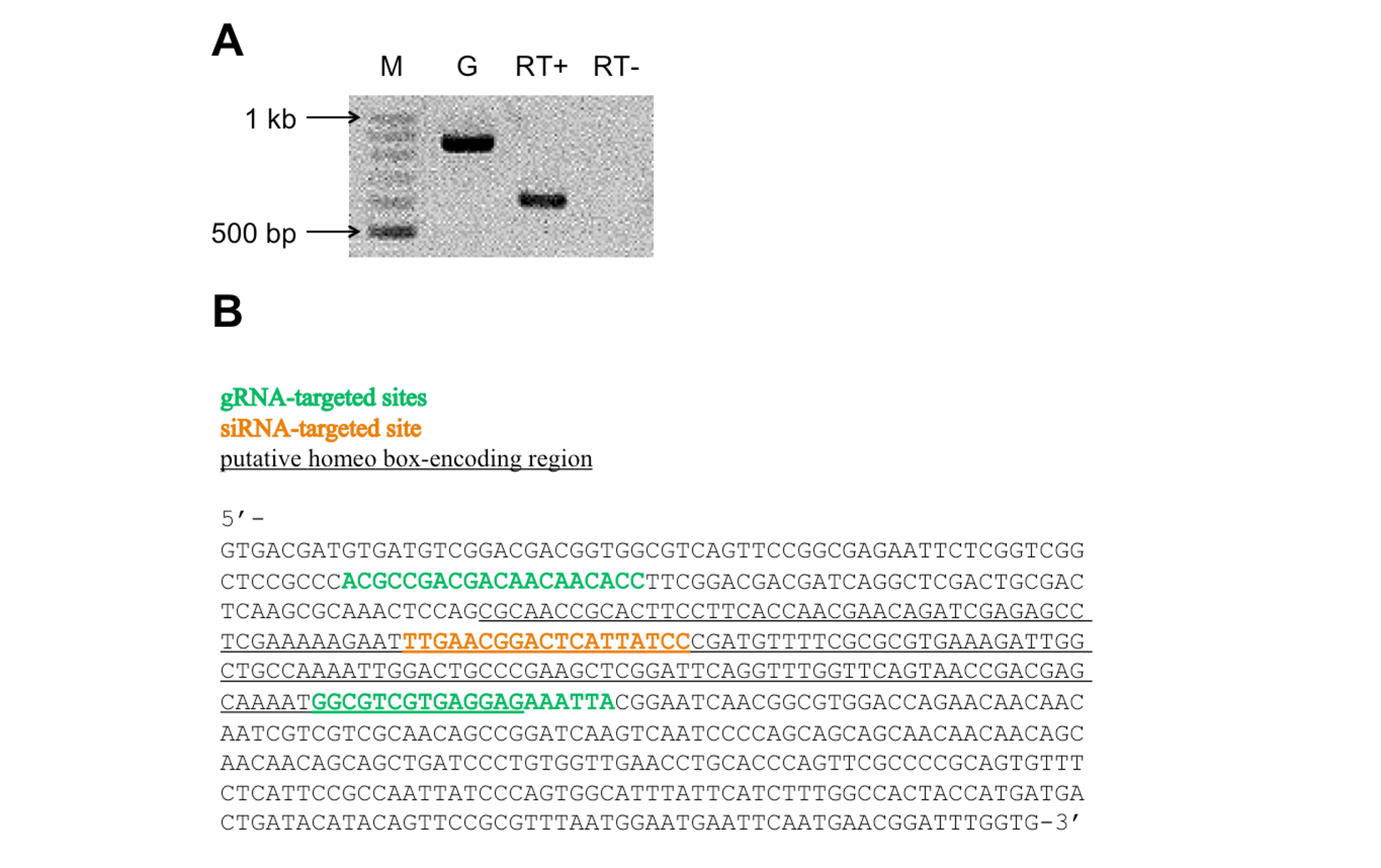

Supplement: Figure S1 — Results of RT-PCR for Dma-ey and cloned partial cDNA sequence. (A) Electrophoresis of PCR products. M: 100-bp ladder marker (TOYOBO, Osaka, Japan), G: genomic PCR product as positive control of PCR, RT+: RT-PCR product amplified from reverse-transcribed cDNAs, RT-: RT-PCR product amplified from total RNAs without reverse transcription. (B) Sequence of partially cloned Dma-ey cDNA. (TIF) [file pone.0098363.s001.tif]
