# Supplementary material for: CRISPR/Cas-Mediated Targeted Mutagenesis in Daphnia magna
Source: PLoS One. 2014 May 30;9(5):e98363. doi: 10.1371/journal.pone.0098363 (PMC4039500; doi:10.1371/journal.pone.0098363)
Supplement: Table S1 — Oligonucleotides for gRNAs and siRNAs. Lowercase “tt” in sense and antisense oligonucleotides for siRNA means dTdT (see Materials and Methods). (DOCX) [file pone.0098363.s002.docx]

**Table S1** Oligonucleotides for gRNAs and siRNAs

| **Names** | **Target sites (5'-3',**  **PAM shown by lowercase)** | **Sense oligonucleotides**  **(5'-3')** | **Antisense oligonucleotides**  **(5'-3')** |
| --- | --- | --- | --- |
| gRNA_Dma-ey_1 | GGTGTTGTTGTCGTCGGCGTggg | TAGGTGTTGTTGTCGTCGGCGT | AAACACGCCGACGACAACAACA |
| gRNA_Dma-ey_2 | GGCGTCGTGAGGAGAAATTAcgg | TAGGCGTCGTGAGGAGAAATTA | AAACTAATTTCTCCTCACGACG |
| Dma-ey_siRNA | TTGAACGGACTCATTATCC | UUGAACGGACUCAUUAUCCtt | GGAUAAUGAGUCCGUUCAAtt |

Lowercase “tt” in sense and antisense oligonucleotides for siRNA means dTdT (see Materials and Methods).
