# Supplementary material for: CRISPR/Cas-Mediated Targeted Mutagenesis in Daphnia magna
Source: PLoS One. 2014 May 30;9(5):e98363. doi: 10.1371/journal.pone.0098363 (PMC4039500; doi:10.1371/journal.pone.0098363)
Supplement: Table S2 — Oligonucleotides used as in vitro transcription templates. (DOCX) [file pone.0098363.s003.docx]

**Table S2 Oligonucleotides used as *in vitro* transcription templates**

| **Names** | **Sequences (5′–3′)** |
| --- | --- |
| Dmavas-5UTR-fwd | GAGACAAAACGTTTCACAATTG |
| Dmavas-5UTRnest-rev | TTTTAAAAGCCTTTTTCAAGTAA |
| Dmavas-3UTR-fwd | CTCGAGGTCGTTAACTTGATTG |
| Dmavas-3UTR-rev | AACAAAATGAATTCGTTCTGTATTC |
| IF-vasMLMCas9-fwd | AAAAGGCTTTTAAAAATGGATAAGAAATACTCAATAG |
| IF-vasMLMCas9-rev | GTTAACGACCTCGAGTCATCCTGCAGCTCCACC |
| IF-pCSvas-fwd | CGAATTCATTTTGTTGGTACCCAGCTTTTGTTCC |
| IF-pCSvas-rev | GAAACGTTTTGTCTCCTCTTCTATAGTGTCACCTAAATCAAG |
| T7-vasCas9-IVT-fwd | TAATACGACTCACTATAGGGAGGAGACAAAAC |
