# Supplementary material for: CRISPR/Cas-Mediated Targeted Mutagenesis in Daphnia magna
Source: PLoS One. 2014 May 30;9(5):e98363. doi: 10.1371/journal.pone.0098363 (PMC4039500; doi:10.1371/journal.pone.0098363)
Supplement: Table S3 — Oligonucleotides used for the verification of cutting sites by sequencing. (DOCX) [file pone.0098363.s004.docx]

**Table S3 Oligonucleotides used for the verification of cutting sites by sequencing**

| **Names** | **Sequences (5′–3′)** |
| --- | --- |
| Dma-ey_homeo_fwd | GTGACGATGTGATGTCGGA |
| Dma-ey_homeo_rev | TCAATGAACGGATTTGGTG |
| Dma-ey_homeo_rev2 | TGCACCACAACAGTCACAAC |
| off855-fwd | GAAATCCAAACAACAAGATTCG |
| off855-rev | GGTGGAGGATACAAAGAACG |
| off915-fwd | GACGCCATCATGTCAACAC |
| off915-rev | CTGCCTGTTAATTCTTCCTCG |
| off986-fwd | TGTTGAGATCCGCTTCAGAC |
| off986-rev | GGAGTGACATCTGACGACTTTG |
| off3390-fwd | AATTCCAAAACACACCCAAG |
| off3390-rev | CGACAAGAACAAACACCACC |
